# Identification of Novel Cholesteatoma-Related Gene Expression Signatures Using Full-Genome Microarrays

**DOI:** 10.1371/journal.pone.0052718

**Published:** 2012-12-20

**Authors:** Christin Klenke, Sebastian Janowski, Daniela Borck, Darius Widera, Jörg Ebmeyer, Jörn Kalinowski, Anke Leichtle, Ralf Hofestädt, Tahwinder Upile, Christian Kaltschmidt, Barbara Kaltschmidt, Holger Sudhoff

**Affiliations:** 1 Department of Otolaryngology and Head and Neck Surgery, Klinikum Bielefeld, Bielefeld, Germany; 2 Bioinformatics Department, University of Bielefeld, Bielefeld, Germany; 3 Cell Biology, University of Bielefeld, Bielefeld, Germany; 4 Department of Otolaryngology and Head and Neck Surgery, University College London Hospital, London, United Kingdom; 5 Molecular Neurobiology, University of Bielefeld, Bielefeld, Germany; 6 Center for Biotechnology, University of Bielefeld, Bielefeld, Germany; National Taiwan University, Taiwan

## Abstract

**Background:**

Cholesteatoma is a gradually expanding destructive epithelial lesion within the middle ear. It can cause extensive local tissue destruction in the temporal bone and can initially lead to the development of conductive hearing loss via ossicular erosion. As the disease progresses, sensorineural hearing loss, vertigo or facial palsy may occur. Cholesteatoma may promote the spread of infection through the tegmen of the middle ear and cause meningitis or intracranial infections with abscess formation. It must, therefore, be considered as a potentially life-threatening middle ear disease.

**Methods and Findings:**

In this study, we investigated differentially expressed genes in human cholesteatomas in comparison to regular auditory canal skin using Whole Human Genome Microarrays containing 19,596 human genes. In addition to already described up-regulated mRNAs in cholesteatoma, such as MMP9, DEFB2 and KRT19, we identified 3558 new cholesteatoma-related transcripts. 811 genes appear to be significantly differentially up-regulated in cholesteatoma. 334 genes were down-regulated more than 2-fold. Significantly regulated genes with protein metabolism activity include matrix metalloproteinases as well as PI3, SERPINB3 and SERPINB4. Genes like SPP1, KRT6B, PRPH, SPRR1B and LAMC2 are known as genes with cell growth and/or maintenance activity. Transport activity genes and signal transduction genes are LCN2, GJB2 and CEACAM6. Three cell communication genes were identified; one CDH19 and two from the S100 family.

**Conclusions:**

This study demonstrates that the expression profile of cholesteatoma is similar to a metastatic tumour and chronically inflamed tissue. Based on the investigated profiles we present novel protein-protein interaction and signal transduction networks, which include cholesteatoma-regulated transcripts and may be of great value for drug targeting and therapy development.

## Introduction

Middle ear cholesteatomas are epidermal inclusion cysts of the middle ear or mastoid. Primary acquired cholesteatomas usually open into the external auditory canal via a ‘defect’ in the tympanic membrane or annulus. They contain desquamated debris comprising mainly keratin from their keratinizing, squamous epithelial lining. The process of pathogenesis is still debated, but cholesteatoma likely arises from the lateral surface epithelium of the tympanic membrane. Middle ear cholesteatomas subsequently grow as a self-perpetuating lesion into the middle ear spaces [1]. Numerous aetiological theories have been proposed; for example the retraction pocket theory, the proliferation theory and immigration theory, as well as the metaplasia theory [2], [3], [4]. In general, cholesteatomas are destructive lesions that gradually expand and lead to complications by destruction of the adjacent structures [5].

Clinically, the onset of disease may be subtle with mild, sometimes intermittent, symptoms. Typically, cholesteatoma produces intermittent unilateral ear discharge, a progressive hearing loss and may be misdiagnosed as recurrent or chronic external otitis or otitis media. Serious complications such as facial palsy, dizziness as well as complete deafness may occur later into the course of the disease. Recent concepts assume that cholesteatoma might be a disturbed wound-healing process [5], often with an underlying inflammatory tissue repair reaction [6]. It has been hypothesized that the development of cholesteatoma involves an altered control of cellular proliferation, which affects the balance towards the aggressive and invasive growth of squamous epithelium [3]. However, it is yet unclear whether this altered control is due to defects in the mechanisms and underlying genes that control proliferation, or to cytokines released from infiltrating inflammatory cells.

The microarray method has been widely used to investigate disease-related changes in gene expression [7], [8], [9]. It was the goal of this study to investigate differential gene expression in human cholesteatoma tissue in comparison to healthy external auditory canal skin using a Human Gene Expression Microarray (HGEM). The expression pattern of cholesteatoma tissue and skin samples was analyzed by HGEM and the expression levels of selected genes were confirmed by real-time PCR and immunohistochemistry.

Up to 811 genes were identified as up-regulated in cholesteatoma tissue with more than 2-fold higher expression compared to healthy skin. In comparison, 334 genes were down-regulated more than 2-fold. Of these genes, the expression values of 33 genes were validated using real-time PCR: 25 of these transcripts displayed differential expression levels between the two tissue types. In 8 cases no differences in the expression were detected. Cytokeratin 6b (KRT6B) and Cytokeratin 14 immunostainings verified increased expression levels in cholesteatoma at protein level. In cholesteatoma matrix metalloproteinases MMP1, MMP9, MMP10, and MMP12 were up-regulated. Interestingly, we found transcription factors including PAX3 and Sp5, TFAP2B to be down-regulated compared to healthy canal skin. To investigate the potential relationship between the differentially expressed genes in human cholesteatoma, we have started to reconstruct protein-protein interaction and signal transduction networks using the software application VANESA.

By using full-genome microarrays and subsequent real-time PCR, we were able to identify several novel cholesteatoma related genes that, after further validation, may be targets for future therapeutic strategies.

## Materials and Methods

### Ethics Statement and human samples

Acquired cholesteatomas and external auditory canal skin specimens were obtained from 17 patients undergoing middle ear surgery at Klinikum Bielefeld Mitte (Bielefeld, Germany). Fully informed written consent was obtained prior to surgery and all clinical investigations were conducted according to the principles of the Declaration of Helsinki (1964) and local guidelines (Bezirksregierung Detmold/Münster).

Both samples (skin and cholesteatoma) were directly placed into liquid nitrogen in the surgical theatre and stored at −80°C prior to RNA extraction.

### RNA amplification labeling and hybridization to Agilent microarrays

The commercially available Whole Human Genome (4x44) Oligo Microarray (Agilent Technologies, Santa Clara, CA, USA) was used in this study according to the instructions of the manufacturer.

RNA was extracted from cholesteatoma and external auditory canal skin using RNasy Mini Kits (Qiagen, Mississauga, ON, Canada) according to manufacturer's instructions. 500 ng of the purified total RNA was subjected to T7-based amplifications using Agilent Amp Labeling Kit to generate fluorescent cRNA. The method uses T7 RNA polymerase, which at the same time amplifies target material and incorporates cyanin 3- or cyanin 5-labeled CTP. Hybridization to whole human genome microarray gene expression chips (Agilent Technologies, Inc., Santa Clara, CA, USA) and dye swaps (Cy3 and Cy5) was performed for RNA, amplified from each specimen. Microarray chips were washed and immediately scanned using a high resolution Agilent© microarray scanner G2565CA (Agilent Technologies, Inc., Santa Clara, CA, USA).

For microarray processing, different Bioconductor software packages (www.bioconductor.org) were used, which provide sophisticated tools for the analysis and comprehension of high-throughput genomic data. Primarily the LIMMA (Linear Models for Microarray Data) [10] package was included in the in-house developed R-analysis pipeline, that uses linear models for the analysis of designed experiments and assessment of differential expression. Its capabilities were used to analyze and investigate the two-color spotted arrays and the two channel microarray experiments.

An essential step in the analysis of the microarray data is the quality check. In order to identify the most significant differentially expressed genes, a background correction was initially performed. This correction subtracts the background intensity from the foreground intensity for each spot. Thus, it was possible to adaptively adjust the foreground from background intensities, which results in strictly positive adjusted intensities [11].

The Loess normalization method for the spotted microarrays was also applied [12]. Without any normalization there can be a considerable variation between both channels and even more between different microarray experiments. Therefore, the M-values for each array were separately normalized, as well as the intensities and log-ratios between the arrays in order to compare them across the different samples. Arrays or samples showing high variations were excluded.

The final process step involved the application of linear models to perform hypothesis tests. This resulted in meaningful (log2) fold changes, standard errors, t-statistics and p-values. Using these statistical calculations the most significant differentially expressed genes within the samples were identified.

Microarray data discussed in this publication have been deposited in NCBI's Gene Expression Omnibus and are accessible through GEO Series accession number GSE42256 (http://www.ncbi.nlm.nih.gov/geo/query/acc.cgi?acc=GSE42256).

### Real-time PCR

Total RNA was extracted from cholesteatoma and skin biopsies using RNeasy Mini Kits (Qiagen, Mississauga, ON, Canada). According to the manufacturer's protocol, 0.5 µg of total RNA was converted to cDNA using the First Strand cDNA Synthesis Kit (Fermentas, St.Leon-Rot, Germany). Following reverse transcription (RT) reaction, all samples were diluted 1∶4 in ddH_2_O and subjected to real time PCR analysis with Maxima SYBR Green qPCR Master Mix (Fermentas, St.Leon-Rot, Germany). 0.3 µM of gene specific primers (Table S1) were used in a total reaction volume of 25 µl. For all targets, the cycling conditions were: 50°C for 2 minutes, 95°C for 10 minutes, followed by 40 cycles each consisting of 95°C for 15 seconds, 60°C for 30 seconds and 72°C for 30 seconds. Integration of SYBR Green dye into the PCR products was monitored using the ABI PRISM 7000 Sequence Detection System (Applied Biosystems, Carlsbad, California). The Pfaffl analysis method was used to measure the relative quantity of gene expression [13]. Serial dilutions were performed to generate a standard curve for each target gene in order to define the efficiency of the qPCR reaction. The specificity of amplified PCR products was confirmed by dissociation curve analysis (SDS software 1.1, Applied Biosystems). The reference gene, GAPDH, was selected based on its stable expression in all tissues analyzed. All measurements were done in triplicates and three independent experiments were performed for each gene target.

### Immunohistochemistry

Cholesteatoma and skin biopsies were fixed using 4% PFA for 60 minutes at 4 °C followed by 3 wash steps in 1xPBS for 5 minutes each. Blocking was done in 5% goat serum for 30 minutes followed by incubation with primary antibodies for 2 hours at room temperature at the following dilutions: rabbit anti-CK5/6 1∶400 (DAKO), rabbit anti-CK14 1∶100 (DAKO). Secondary, fluorochrome-conjugated antibodies were diluted 1∶300 (Alexa 555) and slices were incubated for 1 hour at RT within this solution. Nuclear counterstaining was performed using SYTOX green, 1∶20000 (Molecular Probes) for 30 minutes at RT. The stained sections were mounted with Mowiol (Carl Roth) and dried over night at 4°C. Fluorescence imaging was performed using a confocal microscope (LSM 510, Carl Zeiss, and DM IRB, Leica). Quantitative measurements of the areas of interests were performed using Image J image analysis software (http://rsbweb.nih.gov/ij/). Statistical significance was determined by ANOVA with Bonferroni post hoc test, using GraphPads Prism. P 0.05 was considered significant.

### Bioinformatical network analysis

We used the in-house developed open-source software application VANESA (www.vanesa.sf.net). VANESA aids in modeling experimental results that can be expanded with database information to perform modern biological network analysis [14]. In order to broaden the scope of our model, we also used integrated databases such as HPRD, IntAct and MINT to obtain information of interest and aid network reconstruction.

HPRD is a database of curated proteomic information pertaining human proteins [15]. The provided information is experimentally derived, based on mass spectrometry, protein- microarray, protein-protein interaction, post-translational modifications (PTMs), and tissue expression. A further resource for protein-protein interaction data is the IntAct database [16]. IntAct provides data curated from literature and from direct data deposits. Primarily, it consists of protein-protein interaction data. However, it also includes protein-small molecules for other organisms, such as *Rattus norvegicus*. The Molecular Interaction Database MINT [17] was also queried as it contains approximately 235,000 interactions from over 4,800 publications. MINT contains interactions from more than 30 different species and provides 28,283 interactions for Homo sapiens, 4,808 for *Mus musculus*, and 2,804 entries for *Rattus norvegicus*, which are of great value. A detailed approach of the bioinformatic network analysis based on the mentioned data sources will be published elsewhere (Janowski et al. in preparation).

## Results

### Histology of middle ear cholesteatoma shows a mixture of keratinous material and stratified squamous epithelium

**Figure 1:**
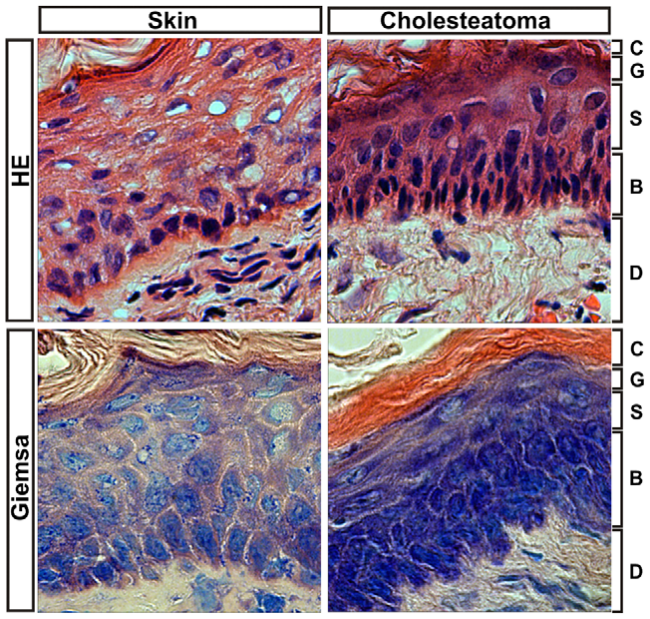
Histology of cholesteatoma. Histologically, a mixture of keratinous material and stratified squamous epithelium is required to diagnose cholesteatoma. The epithelium displays stratified squamous epithelium with keratinization (matrix). The subepithelial region is occupied by connective tissue. Abbreviations: C: stratum corneum; G: stratum granulosum; S: stratum spinosum; B: stratum basale; D: dermis. Magnification, 600x.

Histopathology displayed a mixture of keratinous material and stratified squamous epithelium required for the diagnosis of cholesteatoma (Fig. 1). Cholesteatoma shows bland, keratinizing, stratified squamous epithelium and overlying keratin debris. All examined samples demonstrated the typical histological morphology of cholesteatoma. The epithelium displayed stratified squamous epithelium with keratinization (matrix). The subepithelial region was occupied by connective tissue (Fig. 1). The epithelium of each sample maintained varying thickness (data not shown). The underlying connective tissue presented a variable lympho-plasmocytic infiltrate and appears as an inflammatory network that triggers the cholesteatoma matrix.

### Microarray analysis revealed differential gene expression of transcripts involved in major cellular processes in cholesteatoma

**Figure 2:**
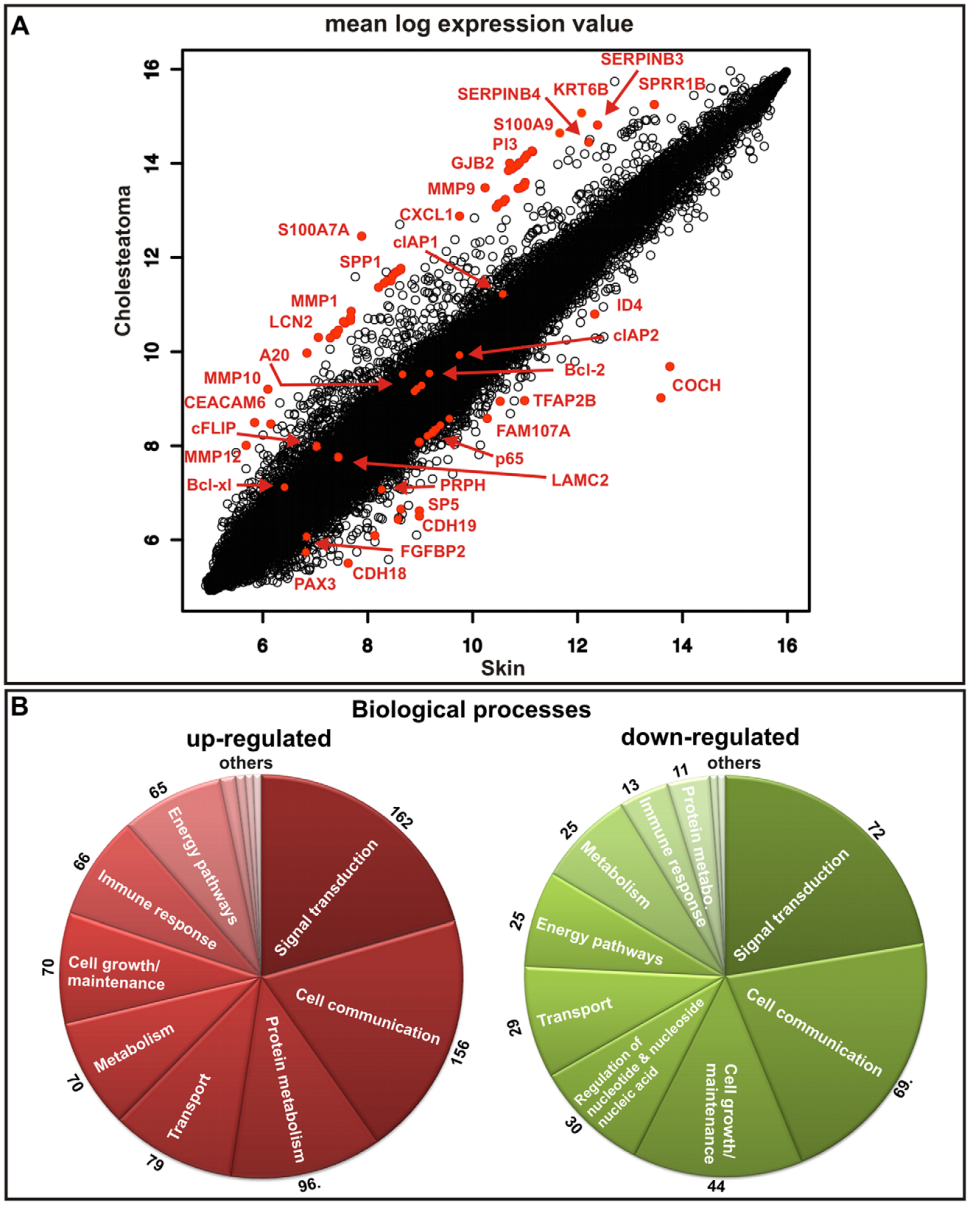
Up-regulated and down-regulated genes in cholesteatoma. (A) mean log expression value plot. The most prominent hits are shown in red. Microarray analysis revealed that the expression of genes, such as PI3, SPRR1B, LCN2 (lipocalin 2), MMP1, MMP9, MMP10, MMP12, SPP1, GJB2, Bcl-xl (BCL2L1), cIAP2 (BIRC3), S100A7A (koebnerisin), S100A9, SERPINB3, CEACAM6, KRT6B and SERPINB4 is higher in cholesteatoma compared to external auditory canal skin. Genes like TFAP2B, CDH18, CDH19, PRPH, ID4, PAX3, LAMC2, SP5 and FGFBP2 were significantly down-regulated. (B) Up-regulated and down-regulated genes in cholesteatoma, according to biological function. Biological processes, which are denoted with ‘others’ are processes with less than 10 genes involved.

To investigate the aeitopathogenesis of cholesteatoma, we examined cholesteatoma tissue from 17 patients undergoing middle ear surgery using a full-genome microarray. After determining the spot intensities at the two wavelengths, a log ratio between the fluorescence intensities was calculated. A total of 811 up-regulated and 334 down-regulated genes in cholesteatoma were identified using 7 selected samples from the microarray data (Figure 2 and Figure S1). These samples were selected using the quality criteria as above. The other samples demonstrated too high variations in the between-array-normalization.

After filtering and data analysis, the following gene groups were identified as demonstrating expression values at least two-fold higher in the cholesteatoma samples compared to the healthy tissue (Figure 2B, Table S2): 162 genes involved in signal transduction, 156 genes in cell communication, 96 genes involved in protein metabolism, 79 genes involved in transport processes, 70 genes related to metabolism, 70 genes related to cell growth and/or maintenance, 66 genes involved in immune response, 65 genes for the energy pathways, 10 relevant genes relevant for the regulation of nucleobase, nucleoside, nucleotide and nucleic acid metabolism, and six apoptosis genes. Biological processes with five or less involved genes are not mentioned and denoted with ‘others’ in Figure 2B.

In contrast, 72 genes involved in signal transduction, 69 genes involved in cell communication, 44 genes involved in cell growth and/or maintenance, 30 genes involved in the regulation of nucleobase, nucleoside, nucleotide and nucleic acid metabolism nucleobase, nucleoside, nucleotide and nucleic acid, 29 genes in transport mechanisms, 25 genes involved in energy metabolism, 25 genes involved in general metabolism, 13 genes involved in immune response and 11 genes involved in protein metabolism were down-regulated (Figure 2B, Table S2). Biological processes which are denoted with ‘others’ in Figure 2B are processes where less than 10 genes are involved.

### Hierarchical clustering of the expression data

**Figure 3:**
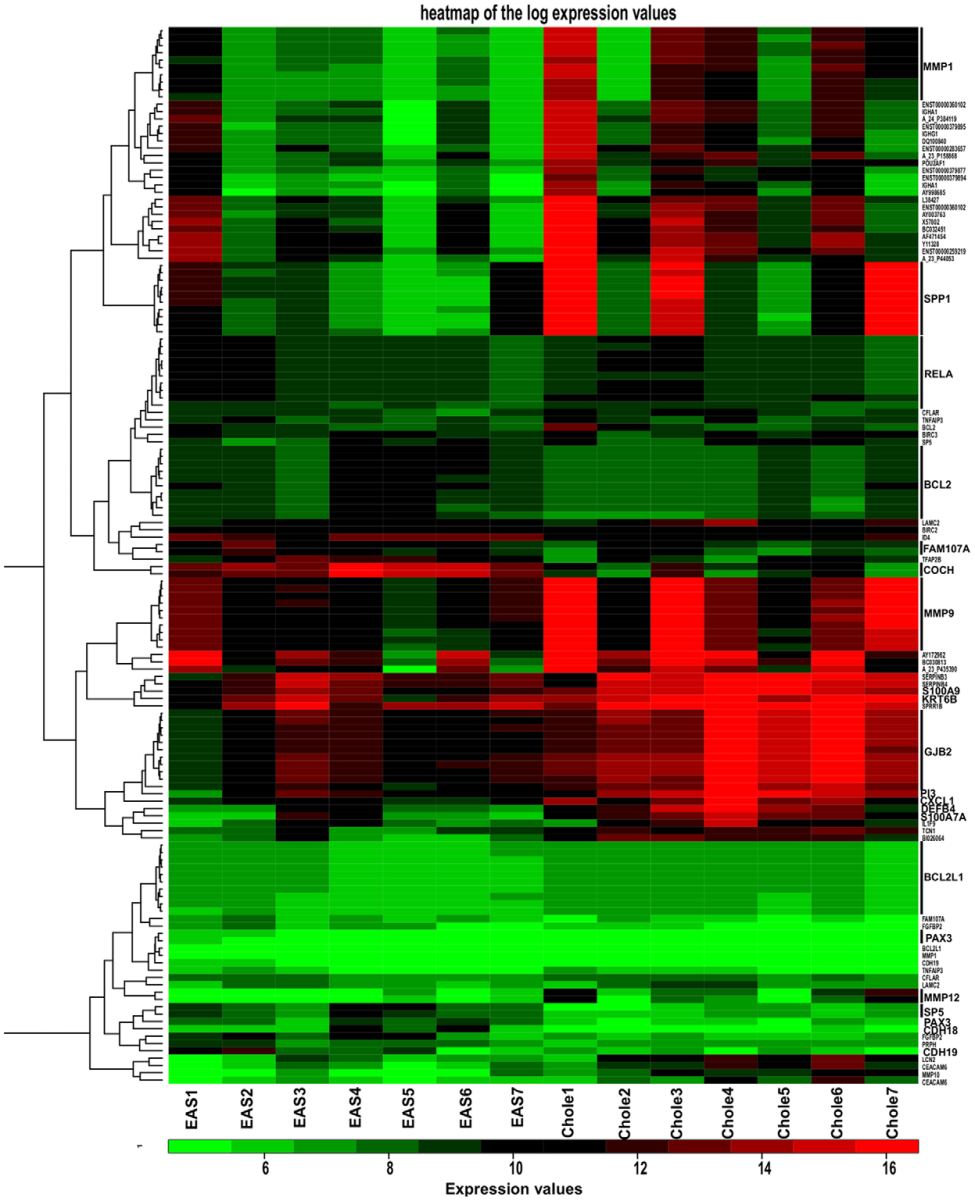
Heatmap of log expression values. For the heatmap generation, significantly regulated genes with a fold change of five were used. 116 up-regulated and 29 down-regulated genes are shown. The clustering reveals that the mRNA profiles of control samples (EAS1-EAS7) show differences to cholesteatoma samples (Chole1-Chole7) relating to the expression of inflammation related genes such as metalloproteinases (e.g. MMP9). The strong co-expression of MMP9 and its known substrate SPP1 (osteopontin 9) might be important. Furthermore, there was a group of patients with low expression of both MMP9 and SPP1, but high expression of genes such as S100A and GJB2.

Hierarchical clustering was conducted by using significantly regulated genes with a fold change over five. In Figure 3, 116 up-regulated and 29 down-regulated genes are shown (Tables S3 and S4). Significantly regulated genes were extracted from datasets and used for hierarchical clustering. Data sets were visualized as heat maps (Fig. 3). Hierarchical clustering reveals that the mRNA profiles of control samples (EAS1-EAS7) differed in comparison to cholesteatoma samples (Chole1-Chole7) concerning the expression of inflammation-related genes such as metalloproteinases (e.g. MMP9). It may be noteworthy to mention the strong co-expression of MMP9 and its known substrate SPP1 (osteopontin 9). There was also a group of patients in which both MMP9 and SPP1 demonstrated low in expression, but these maintained high expression of genes such as S100A and GJB2. Taken together, cluster analysis revealed a highly similar expression pattern of control and cholesteatoma samples.

### Reproducing the microarray data by real-time PCR of representative and significant transcripts

**Figure 4:**
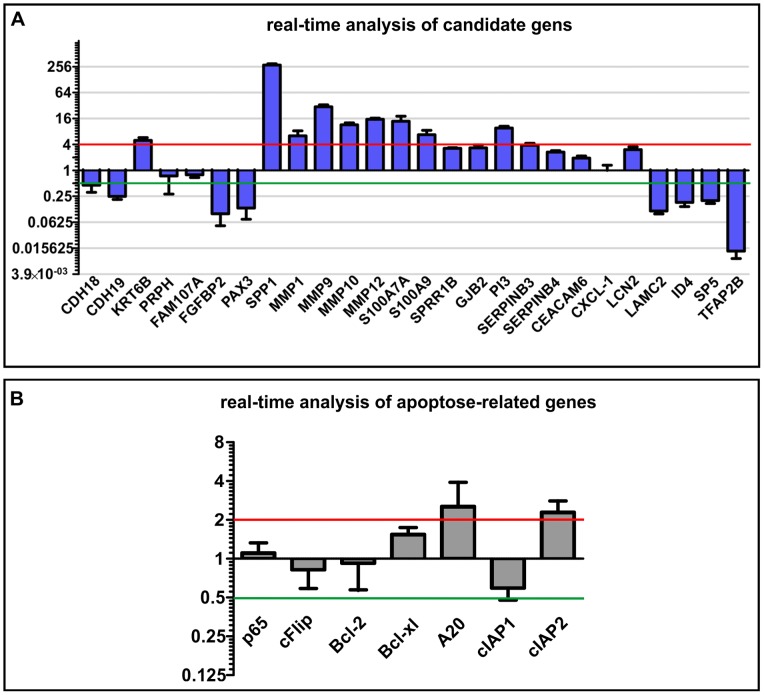
Real-time analysis of candidate genes. (A) The Real-time PCR analysis of potential transcripts that seem to be involved in major processes in cholesteatoma. For normalization the housekeeping gene GAPDH was used. The real-time analysis and comparison of the expression of different genes in external auditory canal skin and cholesteatoma confirms the results of the microarray data. In the present study, genes that encode for MMPs and their substrate were highly up-regulated in cholesteatoma; transcripts that encode e.g. for tumor suppressors were down-regulated. (B) Real-time analysis of apoptosis-related genes. In cholesteatoma genes for the anti-apoptotic proteins Bcl-xl (BCL2L1), A20 (TNFAIP3) and cIAP2 (BIRC3) show a somewhat higher expression. In which cIAP2 (BIRC3) appear to be down-regulated and genes for the proteins p65 (RELA), cFlip (CFLAR) and Bcl-2 show the same expression pattern in cholesteatoma and external auditory skin.

Genes potentially involved in the aforementioned processes in the cholesteatoma samples were analyzed by real-time PCR (Fig. 4). The representative and significantly regulated genes with protein metabolism activity are matrix metalloproteinases as well as PI3, SERPINB3 and SERPINB4. Genes such as SPP1, KRT6B, PRPH, SPRR1B and LAMC2 are known as genes with cell growth and/or maintenance activity. Transport activity genes and signal transduction genes are LCN2, GJB2 and CEACAM6. Three analyzed cell communication genes are CDH19 and other genes belonging to the S100 family. The genes relevant for the regulation of nucleobase, nucleoside, nucleotide and nucleic acid metabolism that seem to be regulated are TFAP2B, ID4, PAX3, among others. SP5 and FGFBP2 as well as CXCL1 have immune response activity. 7 apoptosis-related genes were also analyzed: p65 (RELA), cFlip (CFLAR), Bcl-2, Bcl-xl (BCL2L1), A20 (TNFAIP3), cIAP1 (BIRC2) and cIAP2 (BIRC3)(Fig. 4B).

Real-time PCR using primers designed against message for PI3, SPRR1B, LCN2 (lipocalin 2), MMP1, MMP9, MMP10, MMP12, SPP1, GJB2, Bcl-xl (BCL2L1), cIAP2 (BIRC3), S100A7A (koebnerisin), S100A9, SERPINB3, CEACAM6, KRT6B and SERPINB4 revealed significantly higher expression levels in cholesteatoma in comparison to auditory canal skin. Out of the 334 down-regulated genes, TFAP2B, CDH18, CDH19, PRPH, ID4, PAX3, LAMC2, SP5 and FGFBP2 were analyzed via real-time PCR. The results shown in Figure 4 could verify the expression levels of these genes.

Genes for anti-apoptotic proteins such as Bcl-xl (BCL2L1), A20 (TNFAIP3) and cIAP2 (BIRC3) show only a slightly higher expression in cholesteatoma (logFC 1.5–3) (Fig. 4B). A down-regulation of cIAP1 (BIRC2) was observed, whereas p65 (RELA), cFlip (CFLAR) and Bcl-2 show similar expression levels in cholesteatoma and external auditory skin samples.

### Immunohistochemistry reveals an increased Cytokeratin expression

**Figure 5:**
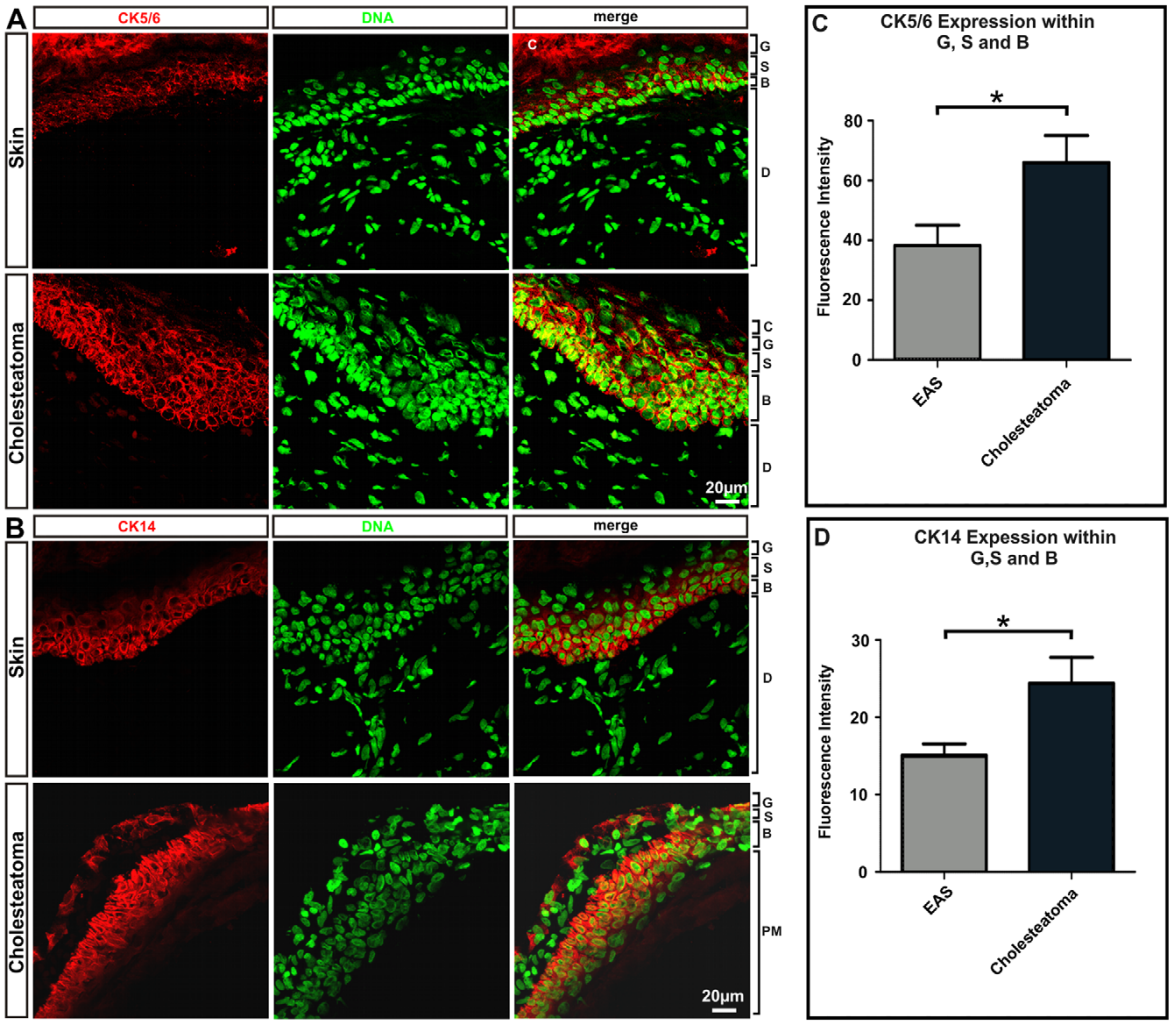
Immunohistochemical staining profiles obtained with CK 5/6 (A) and CK14 (B) antibodies. Using antibodies against Cytokeratin 5/6 and Cytokeratin 14, strong immunoreactivity was detected in the stratum basale and adjacent suprabasal structures, whereas CK5/6 is increased in the pars tensa of cholesteatoma. CK14, which is used as a marker of keratinizing squamous epithelium is synthesized in the basal epithelia cells. (C) Statistical evaluation of CK5/6 expression in cholesteatoma stratum granulosum, stratum spinosum and stratum basale compared to the expression within external auditory skin revealing significantly higher expression in cholesteatoma. p<0.05. (D) Comparison of CK14 expression in cholesteatoma and healthy auditory skin. The basal epithelia cells of cholesteatoma show significantly higher expression of CK14 compared to external auditory skin. p<0,04; Abbreviations: C: stratum corneum; G: stratum granulosum; S: stratum spinosum; B: stratum basale; D: dermis; PM: peri matrix; Magnification: 400x.

Cytokeratins form some of the intermediate filament proteins of epithelial origin and can be used as makers for proliferative keratinocytes [18]. When using antibodies against Cytokeratin 5/6 and Cytokeratin 14, strong immunoreactivity was detected in the stratum basale and adjacent suprabasal structures. CK5/6, a marker of basal keratinocytes and hyper-proliferation [19], is increased in the stratum basale and in the stratum spinosum of cholesteatoma (Fig. 5). The fluorescence signal of CK5/6 is increased by a factor of 1.72 in cholesteatoma epithelium (Fig. 5C). CK14, which is used as a marker of keratinizing squamous epithelium [19], is synthesized in the basal epithelia cells. Figure 5D shows that CK14 is increased by a factor of 1.61.

### Bioinformatical network analysis

As cellular homeostasis is maintained by complex interactions of different regulatory elements, all levels of biological structures can be described as part of a biological network. The smallest level of biological structure is the molecular level; DNA, RNA, proteins, metabolites, and their interactions.

**Figure 6:**
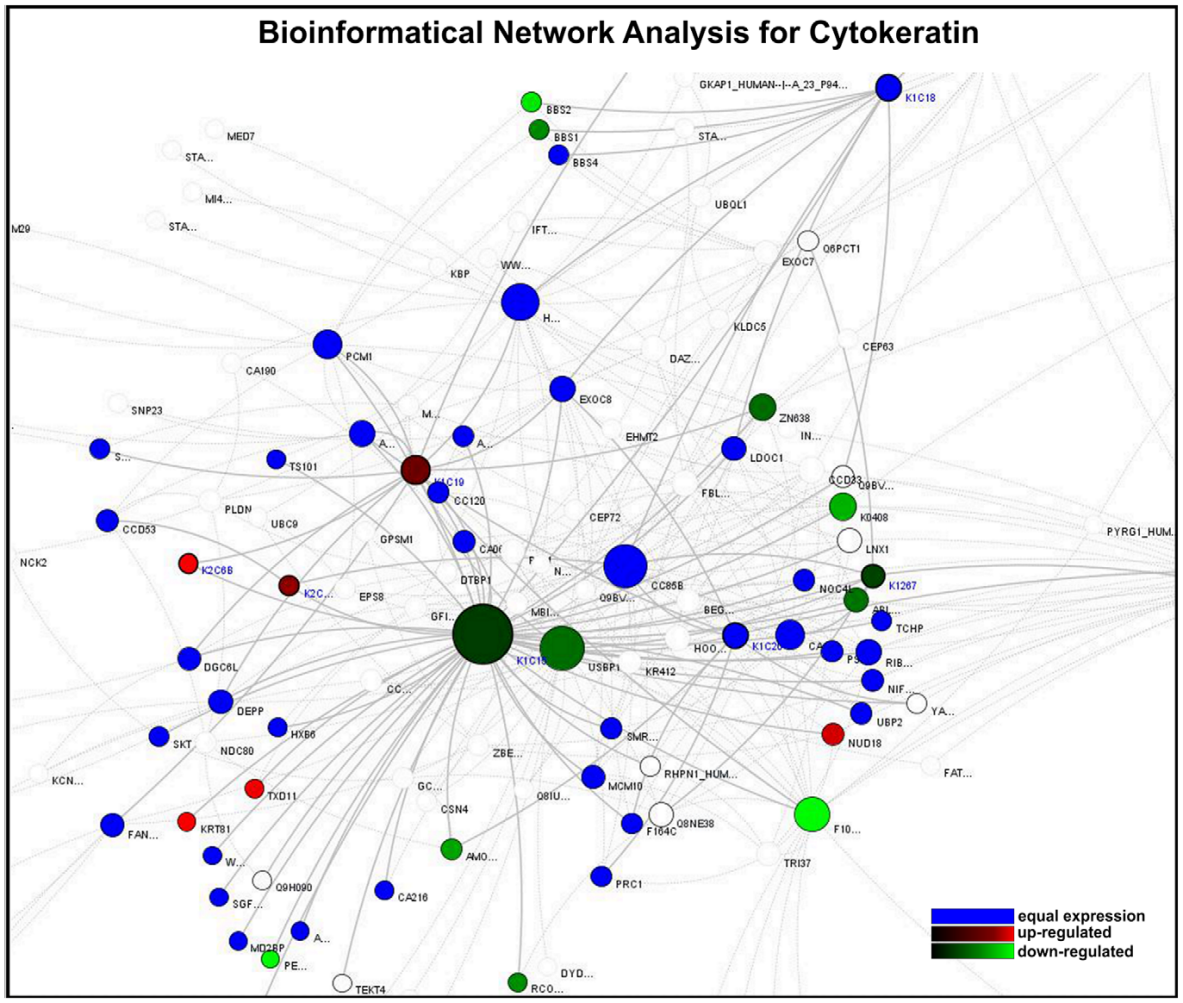
Bioinformatical network analysis for cytokeratin. Proteins are the nodes and the edges are protein activation/inactivations, such as phosphorylation and dephosphorylation across a set of proteins. The cytokeratin analysis reveals an up-regulation of Keratin 6 A and B, Keratin18, Keratin 19 and Keratin 81. In our data, genes for Keratin 15, FAM107A, BBS1 and Peripherin were down-regulated in cholesteatoma. Colors: red: up-reglated genes within cholesteatoma compared to external auditory skin; green: down-regulated genes within cholesteatoma compared to external auditory skin; grey: the gene for the protein is not in the microarray; Node-Size: The “Node Ranking” describes the number of interactions. The bigger the nodes, the more interactions are represented for the protein in the network.

**Figure 7:**
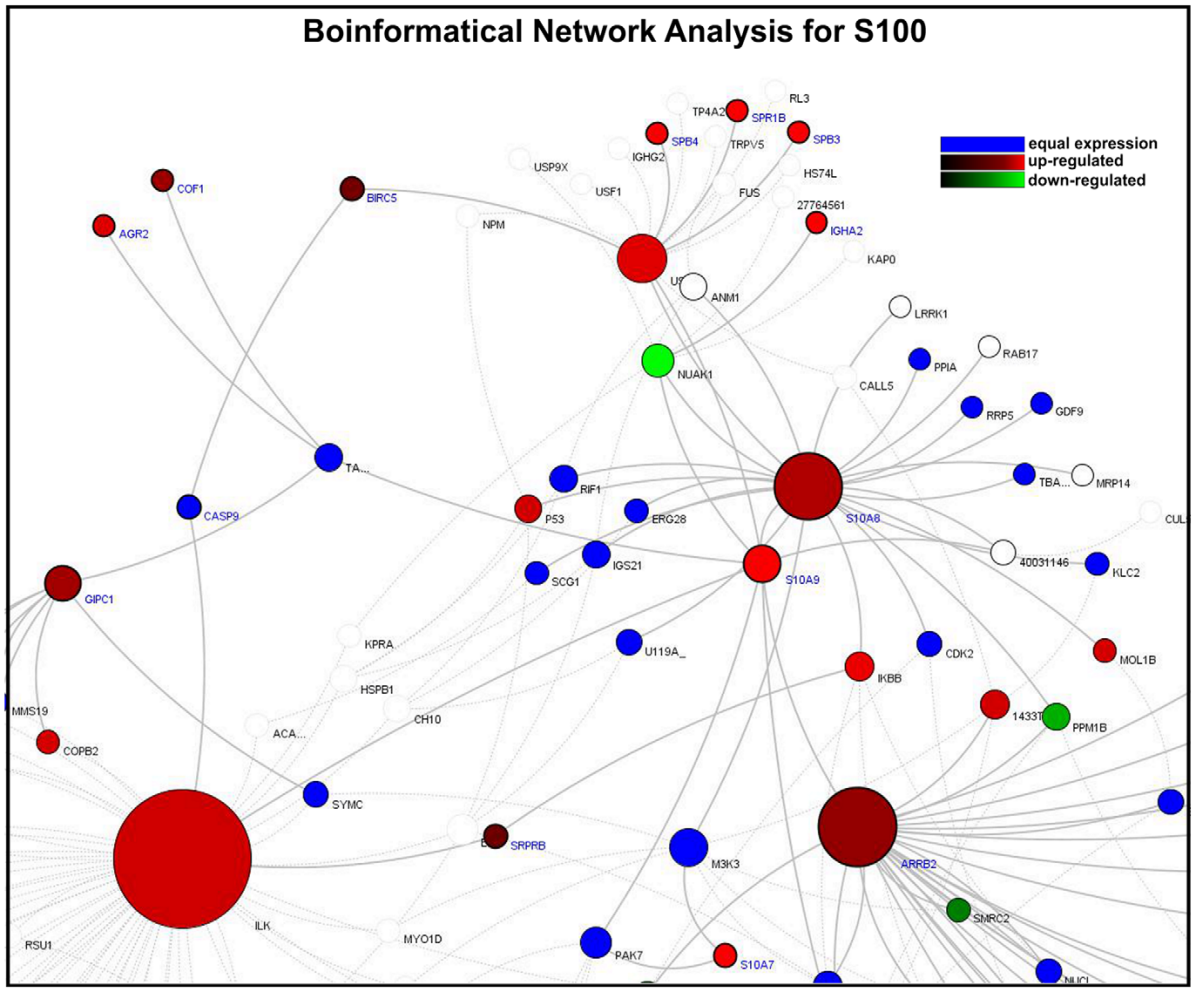
Bioinformatical network analysis for S100. Proteins are the nodes and the edges are protein activation/inactivations, such as phosphorylation and dephosphorylation across a set of proteins. The S100 Network shows an up-regulation of S100A7, S100A8 and S100A9 as well as ILK and IκB, USF2 and ARRB2. In which NUAK1 seems to be down-regulated. Different colors showing the expression of genes with the cholesteatoma compared to external auditory skin: red: up-regulated; green: down-regulated; grey: the gene for the protein is not in the microarray; Node-Size also called “Node Ranking” describes the number of interactions. The bigger the nodes, the more interactions are represented for the protein in the network.

**Figure 8:**
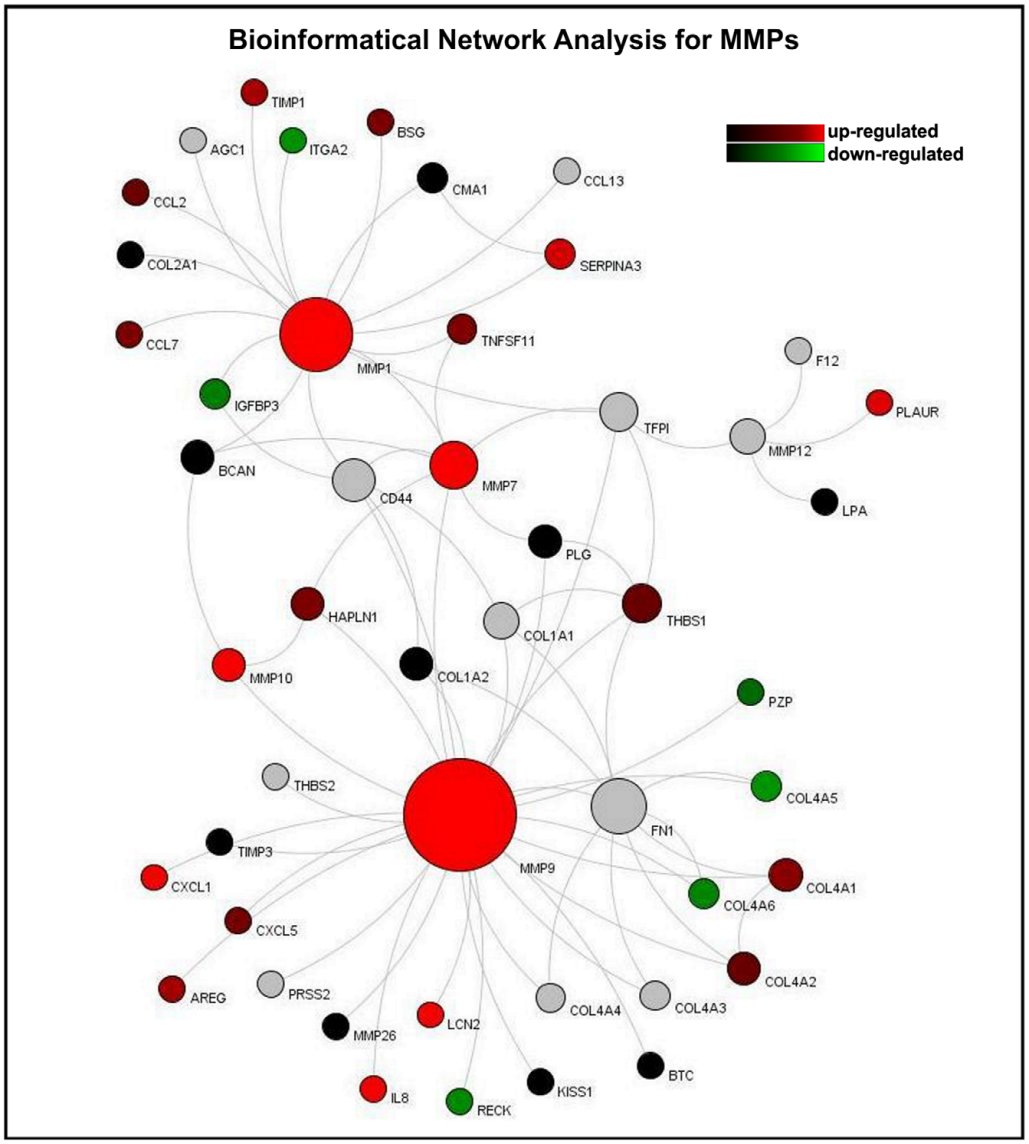
Bioinformatical network analysis for MMPs . Proteins are the nodes and the edges are protein activation/inactivations, such as phosphorylation and dephosphorylation across a set of proteins. The network presented here reveals an up-regulation of the matrix metalloproteinase family members MMP1, 7, 9, 10, 12 as well as LCN2, IL-8 and CXCL1. Down-regulated genes within this network are RECK, COL4A5 and COL4A6. The colors of the nodes describe the regulated expression of genes within cholesteatoma compared to external auditory skin: red: up-regulated genes within cholesteatoma; green: down-regulated genes within cholesteatoma; grey: the gene for the protein is not in the microarray; The “Node Ranking” (Node-Size) describes the number of interactions. The bigger the nodes, the more interactions are represented for the protein in the network.

Based on experimental data and information derived from the integrated databases, we generated model network systems in VANESA. We linked the most significantly expressed microarray results with database content and modeled them as biological interaction networks (see Fig. 6, 7, and 8).

Proteins represent the nodes and the edges denote protein activations/inactivations, such as phosphorylation and dephosphorylation across a set of proteins. The collected data and information is a combination of tightly interlinked complex systems. In particular, the database- generated networks are very complex and difficult to analyze efficiently as the amount of data is vast and multidimensional. Therefore, centrality measurement techniques in VANESA were used to understand the underlying biological processes. Centrality measurement techniques, such as the PageRank and Clustering Algorithm demonstrated diverse functions that are in correlation with the experimental approaches.

Using these analysis methods, the most significant topological skeleton structures of the network could be identified and the prominent actors and connections in cholesteatoma could be identified. Each network is constructed using a few basic mechanistic motifs/modules. The simulated networks demonstrate a dynamic range of target gene activities. The network regulation depends on additional interactions that are clearly visible in so-called multidimensional networks. This results in a set of new, possibly important, regulatory elements and structures that lead to a potential extension of our knowledge of the biological system.

The analysis of the cytokeratin-expression revealed an up-regulation of Keratin 6 A and B, Keratin 18, Keratin 19 and Keratin 81 in cholesteatoma compared to normal skin. In addition, Keratin 15, FAM107A, BBS1 and Peripherin were down-regulated (Fig. 6). Within the S100 network an up-regulation of S100A7, S100A8 and S100A9 as well as ILK and IκB, USF2 and ARRB2 were observed, whereas NUAK1 was down-regulated (Fig. 7). The latter presented network, the MMP network, reveals an up-regulation of the matrix metalloproteinase family members MMP1, 7, 9, 10, 12 as well as LCN2, IL-8 and CXCL1. Down-regulated genes within this network were RECK, COL4A5 and COL4A6 (Fig. 8).

In summary, the genes determined to be up regulated within cholesteatoma indicate many possible networks involved in this disease phenotype. The expression values mapped on the nodes describe the pertubated information and activity flow within the cells. These results provide potential regulatory motifs and paths, which may represent a valuable tool for drug-targeting and therapy after further experimental validation.

## Discussion

The annual incidence of cholesteatoma is around 3 cases in 100.000 children and 9 cases in 100,000 adults [20]. The diagnosis of middle ear cholesteatoma is made on otoscopic examination, including endoscopic and microscopic evaluation as well as surgical exploration. Special imaging procedures, such as high-resolution computed tomography (CT) and magnetic resonance imaging (MRI) may suggest the presence of cholesteatomas within the temporal bone and may be used to complement the clinical examination. Non-EPI diffusion weighted MRI is a valuable addition to the diagnostic tools available to identify cholesteatoma [21]. Acquired cholesteatomas are the consequence of OME or AOM or both. An understanding of the pathogenesis and pathophysiology of aural cholesteatoma is important since the destructive nature of this entity is responsible for much of the morbidity associated with chronic otitis media. The propensity of cholesteatoma to erode bone and the lack of effective, nonsurgical therapy add importance to the understanding of this disease.

Lim and Saunders [22] presented a detailed histology of cholesteatoma in 1972. They demonstrated that cholesteatoma has a keratinized stratified squamous cell epithelium, with layers identical to those in normal skin, Langerhans cells and kerato-hyalin granules (“cholesteatoma matrix”). According to previous descriptions [23], cholesteatomas are covered by keratinizing, stratified squamous epithelium (“matrix”) of varying thickness. These irregularities appear in cholesteatomas derived from different patients, as well as in tissues from the same patient.

By using microarray technology, we were able to identify differentially expressed genes in cholesteatoma compared to healthy external auditory canal skin. Genes like DEFB2 and 3 were not evaluated due to previously published data [7]. Kwon *et al.* showed 2006 that according to gene ontology, 4 cytoskeletal genes, 4 xenobiotic metabolizing enzyme genes, 13 intracellular protein trafficking genes, 19 apoptosis-related genes, 53 signal transduction genes, 14 extracellular matrix/ 19 cell adhesions genes, 7 proteinase inhibitor genes, 58 DNA/RNA binding/ transcription factor genes, 1 calcium homeostatis gene, 8 membran protein genes, 18 cell cycle genes, and 14 immune response genes were up-regulated in cholesteatoma samples when compared to retroauricular skin. To study the gene expression profile in human cholesteatoma Kwon *et al.* used an oligonucleotide chip (Macrogen, Seoul, Korea) including 10115 genes. In contrast, in our approach we used the Agilent-platform (Agilent Technologies, Inc., Santa Clara, CA, USA) including 10596 genes. Microarray results often show variations depending on the technological platform used. Indeed, in our study we were able to show regulation of several genes related to the pathogenesis of cholesteatoma not covered by the study of Kwon et al., such as KRT6B, CEACAM6, SPP1, ID4, PAX3 and RECK. Derived from these findings, we analyzed genes from different processes in more detail: seven genes with protein metabolism activity, five genes with cell growth and/or maintenance activity, two transport activity genes, two signal transduction genes, and three cell communication genes, four genes relevant for the regulation of nucleobase, nucleoside, nucleotide and nucleic acid metabolism and two immune response genes. Seven apoptosis and anti-apoptosis relevant genes were also analyzed. By using real-time PCR analysis, a panel of novel cholesteatoma related genes was verified.

Cytokeratins, like CK6 and CK16 are known as the intermediate filament proteins of epithelial origin and can be used as makers for proliferative keratinocytes [7]. Kwon *et* *al.* reported in 2006 that cytokeratin 1, 13, and 19 are up-regulated in cholesteatoma; interestingly they did not find an up-regulation of cytokeratin 6 or 16 [7]. Cytokeratin expression has been widely found in various malignancies [18]. Using antibodies against Cytokeratin 5/6 and 14, a strong signal was detected in the stratum basale and directly adjacent suprabasal structures [24]. CK14 is synthesized in the basal epithelial cells, and is used as a marker of keratinizing squamous epithelium [18]. Cytokeratin expression observed in the epithelium likely explains proliferative behavior of cholesteatoma, which is linked with increased keratinocyte migration [25]. Keratin 14 is expressed in mitotically active basal layer cells, along with its partner keratin 5, and their expression is down-regulated as cells differentiate [26]. Alam *et al.* showed a reduction of proliferation and a delay in cell cycle progression, along with decreased phosphorylated Akt levels in CK14 knockdown cells [26].

Real-time PCR of PI3, SPRR1B, LCN2 (lipocalin 2), MMP1, MMP9, MMP10, MMP12, SPP1, GJB2, Bcl-xl (BCL2L1), cIAP2 (BIRC3), S100A7A (koebnerisin), S100A9, SERPINB3, CEACAM6, KRT6B and SERPINB4 revealed that the expression levels of these proteins were higher in cholesteatoma than in external canal skin. Proteins of the matrix metalloproteinase (MMPs) family are involved in the breakdown of extracellular matrix; most MMPs are secreted under physiologic conditions. Among the 811 up-regulated extramatrix protein genes, MMP1, MMP7, MMp9, MMP10 and MMP12 and their substrate Osteopontin (SPP1) were amplified and their expression was significantly higher in cholesteatoma than in external canal skin (logFC>10) [27]. It was observed here that the reversion-inducing cystein-rich protein with Kazal motifs *RECK* is significantly down-regulated in cholesteatoma. The RECK gene is extensively expressed in normal tissues, but down-regulated in tumor tissues [28], [29], [30]. It encodes a membrane-anchored glycoprotein and serves as a negative regulator for metalloproteinases [30]. Furthermore *PI3,* which encodes Elafin (also known as peptidase inhibitor 3 or skin-derived antileukoprotease (SKALP)), and its precursor trappin-2, are potent protein inhibitors of neutrophil serine proteases such as leukocyte elastase and proteinase 3 [31]. Alam *et al.* discuss the effects of elafin inhibition of neutrophil elastase and its anti-inflammatory activity [26]. Wei *et al.* evaluated the role of elafin in modulating the sensitivity of human EOC cells to chemotherapeutic drugs [32]. Real-time PCR analysis monitored a 10-fold higher expression of this gene in cholesteatoma compared to skin. Interestingly, genes like Elafin and SPRRs form the cornified envelope typical of squamous cells during squamous metaplasia [33]. Cornifin-B is a protein that is encoded by the *SPRR1B* gene. Its presence correlates with epidermal differentiation in normal skin and skin diseases. Our results indicate that *SPRR1B* expression is up-regulated in cholesteatoma compared to skin (logFC 3). Li *et al.* 2008 and others reported that the synthesis of proteins like SPRRs, loricrin, involcrin, elafin, filaggrin, and keratins results in a change of the appearance of squamous cells. During cholesteatoma development, these cells convert from non-keratinizied, stratified epithelium to a nonsecretory, keratinized epithelium [33].

Lipocalin 2 (LCN2) is known to be highly up-regulated in skin injury and infection events [34]. Lipocalin 2 is an adipokine, whose secretion is highly regulated by activation of inflammation and infection, whereas lipopolysaccharide (LPS) and TNFα are two strong inducers of LCN2 production [35]. The *LCN2* was also verified by real-time PCR, and its expression level is significantly higher when compared to external auditory canal skin (logFC 3). As mentioned above, SPP1 (Osteopontin) serves as a substrate for MMPs and thrombin, it can bind to extracellular matrix proteins (fibronectin and collagen), and is also up-regulated in cholesteatoma (logFC 280 compared to canal skin). Additionally, the message for anti-apoptotic proteins Bcl-xl (BCL2L1), A20 (TNFAIP3) and cIAP2 (BIRC3) show a slightly higher expression in cholesteatoma (logFC 1.5–3) (Fig. 4B). Moreover, we observed down-regulation of cIAP2 (BIRC3). These results correlate with already published results [36]. They indicate that cholesteatoma epithelium demonstrates a similar expression of differentiation and apoptosis-related genes as does normal epithelium [36]. Calcium binding proteins encoded by *S100A7* and *S100A9* revealed a logFC up to 29, both genes are pro-inflammatory genes. The squamous cell carcinoma antigenes SERPINB3 (SCCA1) and SERPINB4 (SCCA2) are circulating tumor markers for squamous cell carcinoma. Their gene expression was also significantly higher in cholesteatoma (logFC 5). CEACAM6 is emerging as an important determinant of the malignant phenotype in a range of cancers [37]; in cholesteatoma this gene is also up-regulated (logFC 2). KRT6B is ontogenically related to cytoskeleton formation.

Out of the 334 down-regulated genes, TFAP2B, CDH18, CDH19, PRPH, ID4, PAX3, LAMC2, SP5 and FGFBP2 were subsequently analyzed via real-time PCR.

We were able to demonstrate that two integral membrane proteins, CDH18 and CDH19, from the cadherin superfamily are down-regulated in cholesteatoma. CDH18 (also known as Cadherin-14) is a novel neutrally specific cell-cell adhesion molecule and may regulate neural morphogenesis. CDH19 (Cadherin-19) is of particular interest as it has been previously shown that it is down-regulated in head and neck cancer cell lines [38]. The ID4 gene encodes an inhibitor of the DNA binding protein family [39]. Solid tumors, including breast, gastric and colon cancer, as well as myelodysplasia are associated with ID4 methylation and silencing [40]. ID4 acts directly as tumor suppressor by influencing cellular processes at multiple levels that lead to a decreased cell proliferation [39]. However, microarray analysis and real-time PCR reveals that this gene is significantly down-regulated in cholesteatoma.

Members of the PAX family play critical roles during fetal development. Alterations in the activity of the *PAX3* gene are associated with some cases of cancer in muscle tissues. *PAX3* has also been recognized as an immunogenic protein in melanomas [41], [42], inducing host immune response against PAX3-expressing tumor cells, resulting in suppression of tumor growth [41]. PAX genes regulate expression of cell surface molecules important for migration [43]. PAX3 is down-regulated in cholesteatoma in both microarray and RT-PCR analysis (Fig 4A). To date, no conclusive case-report in published literature exists and no single case of malignant transformation is yet known. It is, however, known that the AP-2 protein expression levels affect cell transformation, tumor growth and metastasis, and may predict survival in some types of cancer [44]. It has been proposed that the loss of AP-2 is a crucial event in the development of malignant melanoma [44]. The AP-2 transcription factor was found to be down-regulated in cholesteatoma using both microarray and real-time PCR in our analysis. However, the impact of these findings concerning cholesteatoma-pathogenesis should be carefully evaluated in following studies.

Laminins are implicated in a wide range of biological processes including cell adhesion, differentiation, growth, migration, and proliferation [45]. Laminin-5 (LN5) consists of α3, β3 and γ2 chains, which are encoded by LAMA3, LAMB3 and LAMC2 [46]. LN5 plays an important role in epithelial mesenchymal transition through down-regulation of E-cadherin and translocation of β-cadherin into the nuclei [47]. Real-time PCR revealed that LAMC2 is down-regulated in cholesteatomatous tissue.

The expression of Keratin suggests alterations of keratinocyte proliferation, differentiation and migration. In the present study, up-regulation of KRT6 A/B, KRT18, KRT19, KRT81 and down-regulation of KRT15 was identified by microarray and bioinformatic pathway analysis. Immunostainings of cytokeratin revealed increased expression of CK5/6 and CK14. KRT6 and KRT14 are specific to hyper-proliferative conditions of the epidermis and keratinizing squamous epithelium [19]. It is known that KRT15 is down-regulated in hyper-poliferative situations. These results correlate with the opinion that cholesteatoma is a proliferative middle ear disease [18], where keratinocytes express hyperproliferative-associated cytokeratines. After reaching the suprabasal layers they finally undergo apoptosis creating keratinous debris [18].

Interestingly, there is a similarity in the gene expression of cancer/tumor and cholesteatoma (Figure 4 and 6–8). This might explain the aggressive behavior and local destruction of the cholesteatoma mass compared to otitis media, even though the local inflammatory process of chronic otitis media mesotympanalis is similar to that of a cholesteatoma, associated with an induced gene expression in the inflammatory pathways (Leichtle et al, in preparation). Whereas the aggressive behavior of a cholesteatoma mass destroys the surrounding bone and structures regardless of tissue type and may lead to life threatening complications such as hearing loss, facial palsy, meningitis or intracranial abscesses, a behavior which usually is seen in malignant tumors [5].

Some authors postulate the possibility that chronic inflammation may result in a malignant transformation. However, as cholesteatoma is a long-term chronic middle ear disease there are no convincing clinical implications that this postulate is valid. There are no convincing case-reports in the published literature and no single case of malignant transformation was found in our clinical experience (comprises app. 1.800 cases of cholesteatoma over the last 5 years).

In summary, our results suggest that a treatment with metalloproteinase inhibitors (such as doxycycline in a sub anti-microbial dose) perhaps in combination with non-steroidal anti-inflammatory drugs [48] might be a promising supportive treatment option for non-operable cholesteatomas and is the subject of ongoing work.

## Conclusions

In this study, we have demonstrated that the cholesteatoma tissue expresses tumor-relevant genes normally expressed in chronically inflamed tissue. Moreover, tumor suppressor genes CDH18, 19 and ID4, which are known to be down-regulated in various tumors [38], [49], [50] PAX3, LAMC2 and TRAF2B are also down-regulated in cholesteatoma. Genes like CEACA6, MMPs and their inhibitor RECK [30], as well as SPRRB2 are up-regulated.

Genes which were important for inflammation are for example KRT 6 B, SPP1, and S100A7A are highly up-regulated in cholesteatoma compared to external auditory skin. However, further studies should thus evaluate a potential link between inflammation, up-regulation of tumor-related transcripts and cholesteatoma-pathogenesis in more detail.

This is the first report using network analysis on gene expression signatures in cholesteatoma. We present novel protein-protein interaction and signal transduction networks, which include cholesteatoma-regulated transcripts and may be valuable frameworks for drug-targeting and therapy-development.

## Supporting Information

Figure S1 **Normalization of RG densities.** (A) Raw densities of the selected 7 microarray samples. (B) RG densities after normalization within the arrays. (C) RG densities after normalization between all arrays. Summarized: After the Loess normalization of the M-values for each array the red and green distributions become essentially the same, which significantly increases the expressive of the results.
(TIF)

Table S1 **Primers used for real-time PCR analysis.**
(TIF)

Table S2 **Genes related to involved processes.** Listing of significantly up- and down-regulated genes related to involved processes within cholesteatoma compared to external auditory canal skin.
(TIF)

Table S3 **Up-regulated genes.** Listing of significantly up-regulated genes including Gene-Names, Gene-Description, logFC: logarithmic fold change over all experiments, AveExpr: average expression of all average-values, t: T-statistic, P.Value: p-value, adj.P.Val: normalized p-value, and B: log Odds ratio.
(PDF)

Table S4 **Down-regulated genes.** Listing of significantly down-regulated genes including Gene-Names, Gene-Description, logFC: logarithmic fold change over all experiments, AveExpr: average expression of all average-values, t: T-statistic, P.Value: p-value, adj.P.Val: normalized p-value, and B: log Odds ratio.
(PDF)
