# Supplementary figures and images for: Identification of Novel Cholesteatoma-Related Gene Expression Signatures Using Full-Genome Microarrays
Source: PLoS One. 2012 Dec 20;7(12):e52718. doi: 10.1371/journal.pone.0052718 (PMC3527606; doi:10.1371/journal.pone.0052718)

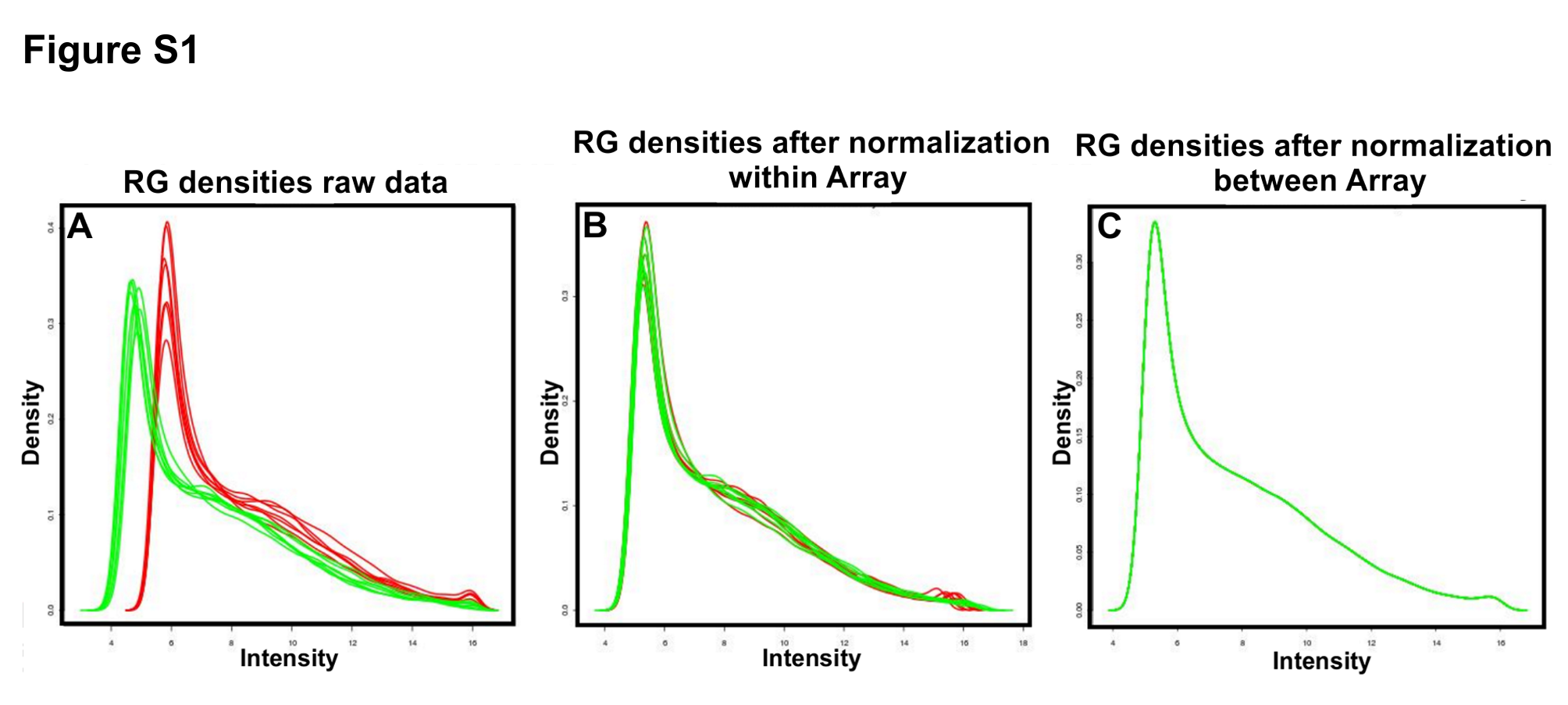

Supplement: Figure S1 — Normalization of RG densities. (A) Raw densities of the selected 7 microarray samples. (B) RG densities after normalization within the arrays. (C) RG densities after normalization between all arrays. Summarized: After the Loess normalization of the M-values for each array the red and green distributions become essentially the same, which significantly increases the expressive of the results. (TIF) [file pone.0052718.s001.tif]

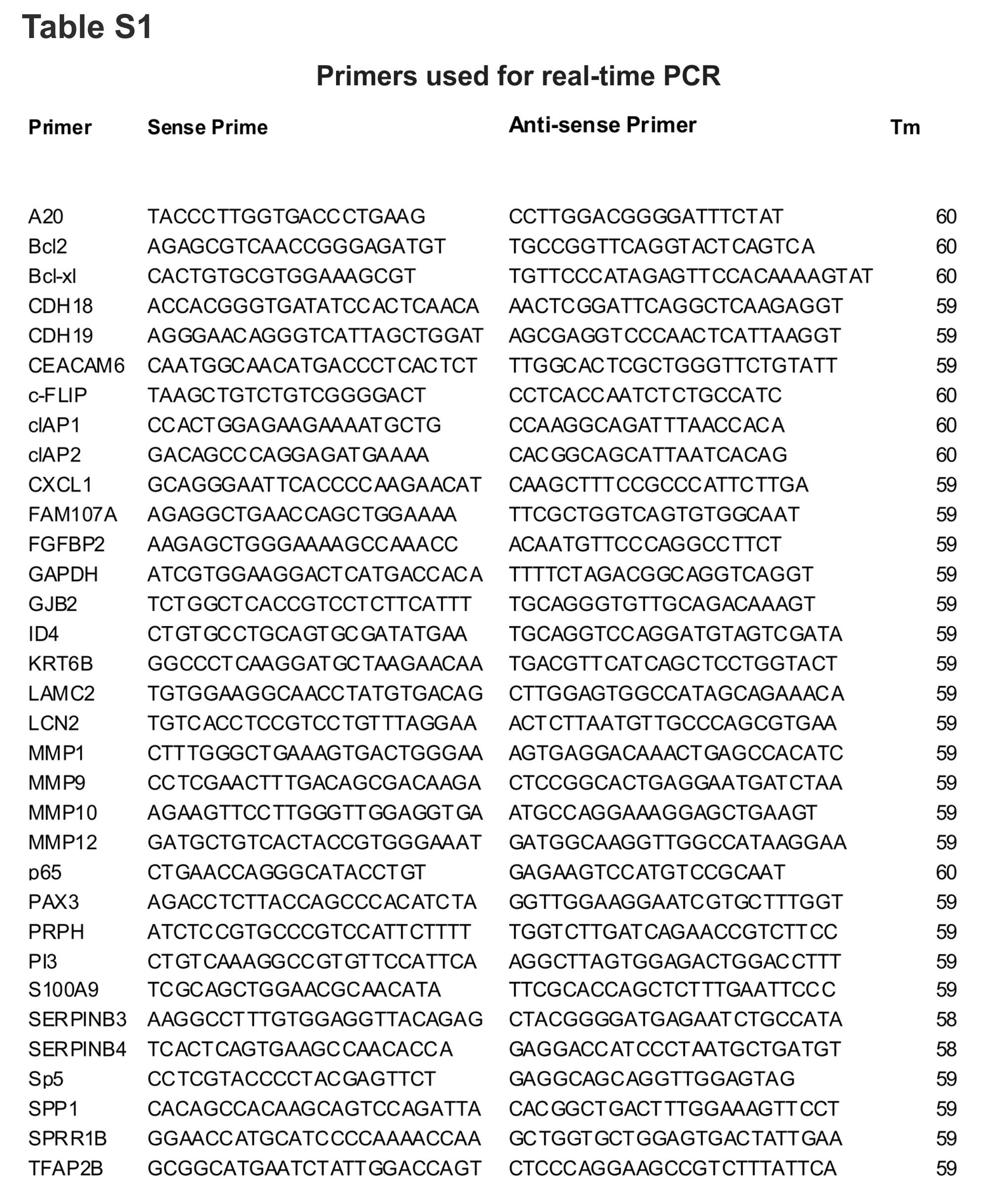

Supplement: Table S1 — Primers used for real-time PCR analysis. (TIF) [file pone.0052718.s002.tif]

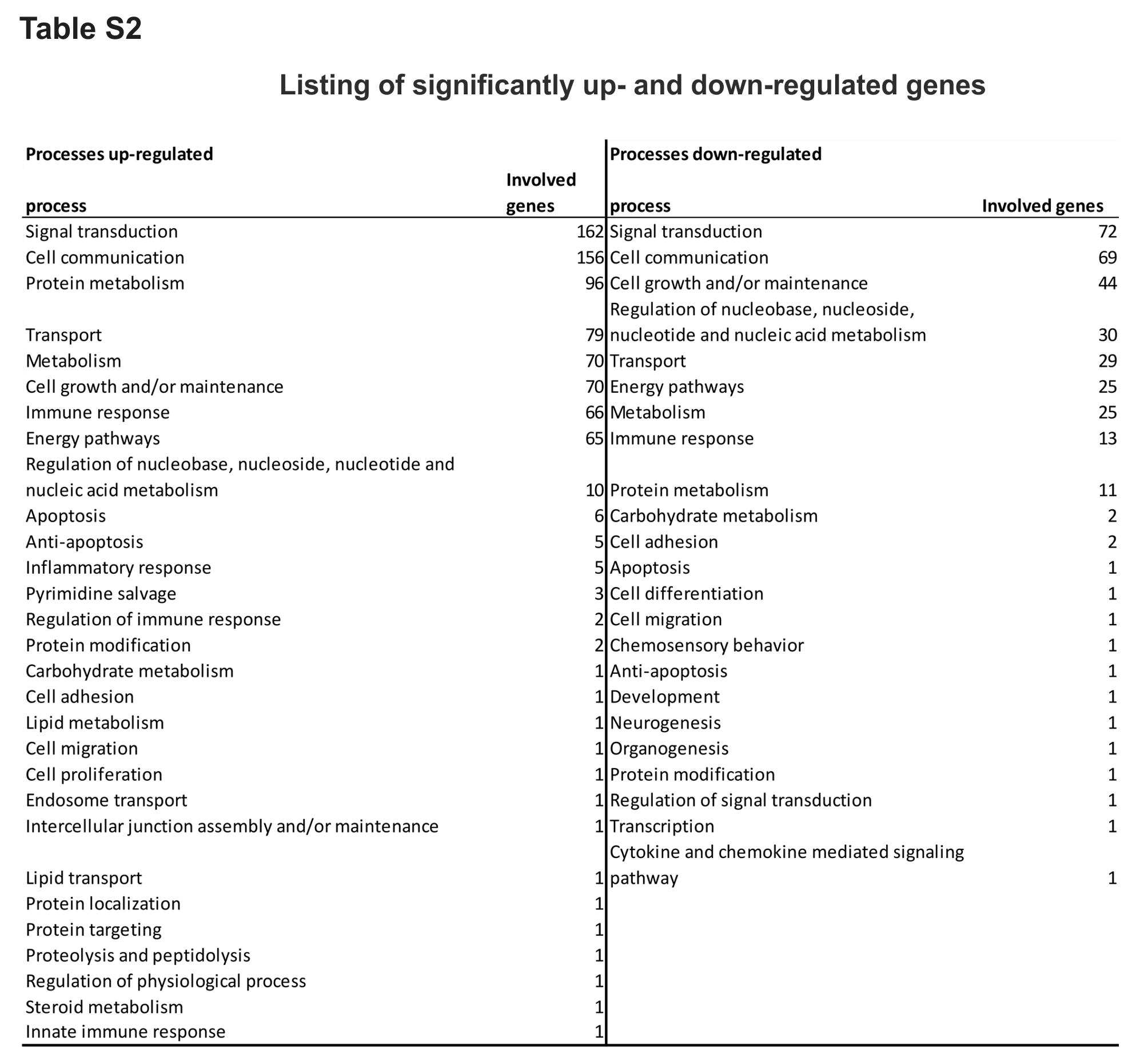

Supplement: Table S2 — Genes related to involved processes. Listing of significantly up- and down-regulated genes related to involved processes within cholesteatoma compared to external auditory canal skin. (TIF) [file pone.0052718.s003.tif]
