# Supplementary material for: Identification of Novel Cholesteatoma-Related Gene Expression Signatures Using Full-Genome Microarrays
Source: PLoS One. 2012 Dec 20;7(12):e52718. doi: 10.1371/journal.pone.0052718 (PMC3527606; doi:10.1371/journal.pone.0052718)
Supplement: Table S3 — Up-regulated genes. Listing of significantly up-regulated genes including Gene-Names, Gene-Description, logFC: logarithmic fold change over all experiments, AveExpr: average expression of all average-values, t: T-statistic, P.Value: p-value, adj.P.Val: normalized p-value, and B: log Odds ratio. (PDF) [file pone.0052718.s004.pdf]

Table S3

up-regulated

GeneName	Description	logFC	AveExpr	t	P.Value	adj.P.Val	B
A_23_P158868	Unknown	2,836187393	9,831630528	10,11936062	8,06E-06	0,005697582	4,271940153
A_23_P435390	Unknown	2,805996935	11,43618933	14,19540928	6,20E-07	0,003489708	6,446510856
A_23_P44053	Unknown	2,584472147	10,13365117	7,156989229	9,89E-05	0,009990662	1,887298413
A_24_P384119	Unknown	2,589793507	9,678541096	10,63166001	5,57E-06	0,005338937	4,603855574
AF471454	Homo sapiens clone 68-46a Ig heavy chain variable region, VH3 family mRNA, partial cds. [AF471454]	2,632473761	10,81334827	11,18755174	3,80E-06	0,005338937	4,942192017
AY003763	Homo sapiens isolate sy-3A/17-G9 immunoglobulin alpha heavy chain variable region mRNA, partial cds. [AY003763]	2,790274559	10,00342781	10,93204781	4,52E-06	0,005338937	4,789392971
AY172962	Homo sapiens anti-rabies SOJB immunoglobulin lambda light chain mRNA, complete cds. [AY172962]	2,412539531	13,06149231	6,881007686	0,000130027	0,010671694	1,616360641
AY998685	Homo sapiens isolate 13K immunoglobulin kappa light chain variable region (IGKV4) mRNA, IGKV4-1*01 allele, partial cds. [AY998685]	2,400738416	8,196804482	6,888318189	0,000129074	0,010661069	1,623662692
BC030813	Homo sapiens cDNA clone MGC:22645 IMAGE:4700961, complete cds. [BC030813]	2,355075139	12,80183102	6,995688186	0,000115934	0,01031523	1,730117924
BC032451	Homo sapiens cDNA clone MGC:40426 IMAGE:5178085, complete cds. [BC032451]	2,366221961	10,36841848	17,98711104	9,92E-08	0,001488983	7,772050492
BCL2	Human B-cell leukemia/lymphoma 2 (bcl-2) proto-oncogene mRNA encoding bcl-2-beta protein, complete cds. [M13995]	0,298614399	9,297452887	1,789519399	0,111512705	0,246646248	-5,166803393
BCL2L1	Homo sapiens BCL2-like 1 (BCL2L1), nuclear gene encoding mitochondrial protein, transcript variant 1, mRNA [NM_138578]	0,362952129	6,903929364	2,900860947	0,019973465	0,085336516	-3,497453858
BCL2L1	Homo sapiens BCL2-like 1 (BCL2L1), nuclear gene encoding mitochondrial protein, transcript variant 1, mRNA [NM_138578]	0,349481987	6,99079181	2,536366094	0,035047734	0,119261935	-4,057416649
		0,347551436	6,97197982	2,789008524	0,023711911	0,094392612	-3,669283243
		0,311927411	6,837171138	2,697283765	0,02731377	0,102751912	-3,810305969
		0,310576797	6,949719123	2,130043346	0,06596259	0,175615446	-4,672990821
		0,307047096	6,909913873	2,406740442	0,042876527	0,134510201	-4,255607621
		0,298554304	6,966415889	2,109725881	0,068077766	0,179089809	-4,703192427
		0,281001848	6,737902565	1,973240246	0,084105987	0,20576718	-4,903920608
		0,257240956	6,7116457	2,056501543	0,073938793	0,189211574	-4,781936868
		0,242026062	6,654352487	1,646343962	0,138503011	0,284651099	-5,364222563
BCL2L1	Homo sapiens BCL2-like 1 (BCL2L1), nuclear gene encoding mitochondrial protein, transcript variant 2, mRNA [NM_001191]	0,060620726	5,390451813	0,772807632	0,461966352	0,622154149	-6,333592941
BI026064	BI026064 CM0-MT0374-060201-774-h11 MT0374 Homo sapiens cDNA, mRNA sequence [BI026064]	3,824480254	9,675470633	7,203959216	9,45E-05	0,00989597	1,932457933
BIRC3	Homo sapiens baculoviral IAP repeat-containing 3 (BIRC3), transcript variant 1, mRNA [NM_001165]	0,205488323	9,742221803	0,925606854	0,381857835	0,549759504	-6,202129391

CEACAM6	Homo sapiens carcinoembryonic antigen-related cell adhesion molecule 6 (non-specific cross reacting antigen), mRNA (cDNA clone MGC:10467 IMAGE:3640231), complete cds. [BC005008]	3,130636354	8,406677007	4,836358799	0,001312956	0,021604719	-0,721328819
CEACAM6	Homo sapiens carcinoembryonic antigen-related cell adhesion molecule 6 (non-specific cross reacting antigen) (CEACAM6), mRNA [NM_002483]	2,311365801	7,308213914	3,948968603	0,004285435	0,03718693	-1,933228674
CFLAR	Homo sapiens FLAME-1 mRNA, complete cds. [AF009616]	0,81094224	9,105712037	6,884020061	0,000129634	0,010671694	1,619370369
CFLAR	Homo sapiens CASP8 and FADD-like apoptosis regulator (CFLAR), mRNA [NM_003879]	0,488736648	7,607799379	4,684019654	0,001595394	0,023251299	-0,920773375
CXCL1	Homo sapiens chemokine (C-X-C motif) ligand 1 (melanoma growth stimulating activity, alpha) (CXCL1), mRNA [NM_001511]	3,12959711	11,3143869	4,967570941	0,001113111	0,020182113	-0,552456472
DEFB4	Homo sapiens defensin, beta 4 (DEFB4), mRNA [NM_004942]	4,092558128	10,65623526	9,390967016	1,40E-05	0,006520059	3,763728772
DQ100840	Homo sapiens isolate N1553H immunoglobulin heavy chain variable region (IGHV3-21) mRNA, IGHV3-21*01 allele, partial cds. [DQ100840]	2,328777777	9,114283866	7,111811462	0,000103367	0,010010246	1,843603651
ENST00000259219	Homo sapiens clone 63a12 anti-tetanus toxoid immunoglobulin light chain variable region (IGL@) mRNA, partial cds. [AY867113]	2,716544981	11,0201082	8,010383427	4,45E-05	0,007930907	2,66691849
ENST00000283657	V kappa 4=immunoglobulin light chain variable region {complementarity determining regions} [human, CD5+ tonsillar B cells, mRNA PartialMutant, 303 nt]. [S62210]	2,483016726	9,478342955	14,79035224	4,52E-07	0,003489708	6,690113458
ENST00000360102	Homo sapiens clone CD-27-VH immunoglobulin heavy chain variable region mRNA, partial cds. [AY944711]	2,644248462	10,21603904	9,275101373	1,54E-05	0,006520059	3,678685907
ENST00000360102	AF062202 immunoglobulin heavy chain variable region {Homo sapiens} (exp=-1; wgp=0; cg=0), partial (93%) [THC2579601]	2,438297497	9,472277229	8,24854474	3,61E-05	0,007440784	2,869832288
ENST00000379877	Full-length cDNA clone CS0DL004YM19 of B cells (Ramos cell line) of Homo sapiens (human) (Fragment). [Source:Uniprot/SPTREMBL;Acc:Q86SX2] [ENST00000379877]	2,555988918	8,964201718	8,388778091	3,19E-05	0,0072247	2,98650131
ENST00000379894	Ig heavy chain V-II region ARH-77 precursor. [Source:Uniprot/SWISSPROT;Acc:P06331] [ENST00000379894]	2,404193267	8,364427565	8,933657966	2,02E-05	0,006707035	3,420956523
ENST00000379895	Immunglobulin heavy chain variable region (Fragment). [Source:Uniprot/SPTREMBL;Acc:Q0ZCI9] [ENST00000379895]	2,464440424	9,184232537	10,06947379	8,36E-06	0,005790337	4,238530457
GJB2	Homo sapiens gap junction protein, beta 2, 26kDa (connexin 26) (GJB2), mRNA [NM_004004]	3,246038519	11,85909721	6,493697633	0,000193627	0,011695552	1,219451523
		3,16599253	12,26498479	7,003388751	0,00011505	0,010296206	1,737696316
		3,145669301	12,60318583	7,2143572	9,35E-05	0,00989597	1,942418286
		3,135569195	12,69728852	7,407717952	7,77E-05	0,009699687	2,125237306
		3,135456521	12,3138536	7,224663863	9,26E-05	0,009876193	1,952278027
		3,131397339	12,35514995	7,354192603	8,17E-05	0,009699687	2,07508333
		3,124273737	12,44975284	7,240975751	9,11E-05	0,009797466	1,967855912
		3,121923374	12,45040368	7,294741229	8,65E-05	0,009741322	2,01897146
		3,116471231	12,54551339	7,10814744	0,00010374	0,010010246	1,840048746
		3,112766985	12,40569108	7,36362221	8,10E-05	0,009699687	2,083943994
		3,108299663	12,69505213	7,359452852	8,13E-05	0,009699687	2,08002752

IGHA1	Homo sapiens cDNA FLJ46621 fis, clone TLUNG2001445, highly similar to Ig alpha-1 chain C region. [AK128476]	2,553263729	9,404915748	7,138633416	0,000100684	0,010006798	1,869575802
IGHA1	Homo sapiens SNC73 protein (SNC73) mRNA, complete cds. [AF067420]	2,515677273	8,458890448	6,780940545	0,000143885	0,010872777	1,515712267
IGHG1	Homo sapiens immunoglobulin heavy constant gamma 1 (G1m marker), mRNA (cDNA clone MGC:105004 IMAGE:3056327), complete cds. [BC092518]	2,537392243	9,096562685	9,813819369	1,01E-05	0,006362644	4,064160164
IL1F9	Homo sapiens interleukin 1 family, member 9 (IL1F9), mRNA [NM_019618]	2,527728568	9,678555718	6,241316994	0,000253333	0,012490268	0,949887701
KRT6B	Homo sapiens keratin 6B (KRT6B), mRNA [NM_005555]	2,996476877	13,57401676	7,910273471	4,87E-05	0,008153463	2,579787278
L38427	Homo sapiens Ig rearranged H-chain mRNA V region. [L38427]	2,43176799	10,31500896	8,914184777	2,05E-05	0,006707035	3,405929536
LAMC2	Homo sapiens laminin, gamma 2 (LAMC2), transcript variant 2, mRNA [NM_018891]	0,728625961	7,45782842	2,856770783	0,021368366	0,088658461	-3,565151659
LAMC2	Homo sapiens laminin, gamma 2 (LAMC2), transcript variant 1, mRNA [NM_005562]	0,618612708	10,89802232	2,074219142	0,071934975	0,185673904	-4,755786185
LCN2	Homo sapiens lipocalin 2 (oncogene 24p3) (LCN2), mRNA [NM_005564]	3,247618542	8,681488609	5,78557906	0,000419793	0,014336694	0,440238337
MMP1	Homo sapiens matrix metallopeptidase 1 (interstitial collagenase) (MMP1), mRNA [NM_002421]	3,170929542	9,269180174	4,541024276	0,001921486	0,025352464	-1,111282167
		3,098470566	9,087027629	4,564712338	0,001862804	0,025013101	-1,079503354
		3,05373649	9,199989428	4,506526541	0,002010567	0,025925433	-1,157718353
		3,047701424	9,106770011	4,529438406	0,001950916	0,025544618	-1,126856936
		3,041160237	9,086729891	4,557544265	0,001880353	0,025129116	-1,08911052
		3,022662297	8,879314228	4,497319409	0,002035093	0,026072061	-1,170142895
		3,019149154	8,949854534	4,583423137	0,001817827	0,024660537	-1,054463412
		3,008614077	8,789563459	4,561115782	0,001871587	0,025046704	-1,084322719
		2,992202842	9,166590084	4,44171598	0,002190282	0,02691075	-1,245454999
		2,966466971	8,884212273	4,528466507	0,001953407	0,025545237	-1,128164396
		0,161140859	5,355019907	3,568506178	0,007370909	0,049287109	-2,48760403
MMP10	Homo sapiens matrix metallopeptidase 10 (stromelysin 2) (MMP10), mRNA [NM_002425]	3,105657726	7,649534038	10,75251532	5,12E-06	0,005338937	4,679285426
MMP12	Homo sapiens matrix metallopeptidase 12 (macrophage elastase) (MMP12), mRNA [NM_002426]	2,650468819	7,169162009	3,311545277	0,01075061	0,060341733	-2,87178434
		2,325773134	6,844968403	3,288760872	0,011120966	0,061452291	-2,906169384
MMP9	Homo sapiens matrix metallopeptidase 9 (gelatinase B, 92kDa gelatinase, 92kDa type IV collagenase) (MMP9), mRNA [NM_004994]	2,644116775	11,81686806	4,836096318	0,001313393	0,021604719	-0,72166934
		2,616804714	11,7582809	4,856837601	0,001279342	0,021416735	-0,694794694
		2,610722767	11,92697104	4,937990995	0,001155077	0,020510945	-0,590291066
		2,60007894	12,16653436	4,938161718	0,00115483	0,020510945	-0,590072308
		2,596011	11,88136198	4,765976429	0,001436025	0,022369166	-0,813023086
		2,595409746	12,29627101	4,910418455	0,001195754	0,020804804	-0,625681381
		2,570536642	12,19054981	4,902928302	0,001207072	0,020915753	-0,635315811
		2,566032736	12,24732239	4,868383894	0,001260805	0,02129453	-0,679863264
		2,558870866	12,24727348	4,912543334	0,001192564	0,020804804	-0,622949792
		2,550837325	12,2275991	4,883123116	0,001237566	0,021109901	-0,66083314

PI3							
	Homo sapiens peptidase inhibitor 3, skin-derived (SKALP) (PI3), mRNA [NM_002638]	3,298747876	12,35451416	8,396090991	3,17E-05	0,0072247	2,99252935
POU2AF1	Homo sapiens POU domain, class 2, associating factor 1 (POU2AF1), mRNA [NM_006235]	2,321902482	9,545086738	14,63243761	4,91E-07	0,003489708	6,626936523
RELA							
	Homo sapiens v-rel reticuloendotheliosis viral oncogene homolog A, nuclear factor of kappa light polypeptide gene enhancer in B-cells 3, p65 (avian), mRNA (cDNA clone MGC:131774 IMAGE:6019711), complete cds. [BC110830]	0,203050269	9,397286683	1,752547188	0,117967769	0,256112645	-5,218476548
		0,170639269	9,170383376	1,444775914	0,186711018	0,345387675	-5,628063823
		0,169093196	9,174137115	1,622673565	0,143508897	0,291306381	-5,396120486
		0,168425183	9,325171383	1,385950138	0,20335368	0,364772501	-5,70140942
		0,164471794	9,309896601	1,465750079	0,181074097	0,338470663	-5,601485769
		0,162803751	9,22377173	1,363868218	0,209927254	0,372579486	-5,728469085
		0,160594029	9,202688853	1,395955602	0,200434574	0,36164024	-5,689062095
		0,159660134	9,232497974	1,554252661	0,158922971	0,311581774	-5,48701606
		0,147530701	8,835071521	1,431247625	0,19042855	0,349569414	-5,645089526
		0,11199754	9,060744072	0,948025754	0,371007192	0,539382126	-6,181221992
S100A7A							
	Homo sapiens S100 calcium binding protein A7A (S100A7A), mRNA [NM_176823]	4,568631685	10,16747443	9,232064882	1,59E-05	0,006561081	3,646791041
S100A9							
	Homo sapiens S100 calcium binding protein A9 (S100A9), mRNA [NM_002965]	2,984552635	13,15252485	7,564631618	6,70E-05	0,009508635	2,270302186
SERPINB3	Homo sapiens serpin peptidase inhibitor, clade B (ovalbumin), member 3 (SERPINB3), mRNA [NM_006919]	2,434148264	13,59728663	5,876810887	0,00037863	0,013947646	0,544677892
SERPINB4	Homo sapiens serpin peptidase inhibitor, clade B (ovalbumin), member 4 (SERPINB4), mRNA [NM_002974]	2,238709793	13,33183614	5,552200441	0,000549331	0,015680502	0,167435443
SP5	Homo sapiens Sp5 transcription factor (SP5), mRNA [NM_001003845]	0,492798859	9,424463969	3,474057467	0,00845932	0,053163987	-2,62799547
SPP1							
	Homo sapiens secreted phosphoprotein 1 (osteopontin, bone sialoprotein I, early T-lymphocyte activation 1) (SPP1), transcript variant 1, mRNA [NM_001040058]	3,155514466	9,784187957	4,008269155	0,003945428	0,035742289	-1,848574146
		3,152415907	10,09361125	4,153569049	0,003229038	0,032058924	-1,643284836
		3,144480696	10,12014791	4,105260767	0,003450292	0,033236666	-1,711197245
		3,14011476	9,881204363	4,094777291	0,003500433	0,033468988	-1,725980173
		3,138414705	10,19839688	4,072088119	0,00361166	0,034007092	-1,758029329
		3,136831774	10,03627911	4,141507499	0,003282809	0,032323603	-1,660209004
		3,136090697	10,13166768	4,155168243	0,003221981	0,032013266	-1,641042537
		3,128357977	9,938744019	4,088503785	0,003530813	0,033599711	-1,734834211
		3,110705791	10,18282331	4,037932732	0,003786337	0,034976799	-1,806415381
		3,061361235	9,971307841	3,878783587	0,004729024	0,039056395	-2,034056708
SPRR1B							
	Homo sapiens small proline-rich protein 1B (cornifin) (SPRR1B), mRNA [NM_003125]	1,788611858	14,35666836	3,968558215	0,004169756	0,036768186	-1,905208616

TCN1	Homo sapiens transcobalamin I (vitamin B12 binding protein, R binder family) (TCN1), mRNA [NM_001062]	3,336643959	10,02192092	7,441314285	7,52E-05	0,009678162	2,15654225
TNFAIP3	Homo sapiens tumor necrosis factor, alpha-induced protein 3 (TNFAIP3), mRNA [NM_006290]	0,289548006	8,943257819	0,976175216	0,357709664	0,527348745	-6,154410387
		0,192122173	5,981729566	1,212696514	0,260008958	0,428744762	-5,906212262
X57802	Human rearranged immunoglobulin lambda light chain mRNA. [X57802]	2,690326729	10,44100328	9,135803295	1,72E-05	0,006707035	3,574840608
Y11328	H.sapiens mRNA for immunoglobulin heavy chain, partial, clone VH3-7. [Y11328]	2,696710741	10,61500144	11,11736361	3,99E-06	0,005338937	4,900665997
