# Supplementary material for: Identification of Novel Cholesteatoma-Related Gene Expression Signatures Using Full-Genome Microarrays
Source: PLoS One. 2012 Dec 20;7(12):e52718. doi: 10.1371/journal.pone.0052718 (PMC3527606; doi:10.1371/journal.pone.0052718)
Supplement: Table S4 — Down-regulated genes. Listing of significantly down-regulated genes including Gene-Names, Gene-Description, logFC: logarithmic fold change over all experiments, AveExpr: average expression of all average-values, t: T-statistic, P.Value: p-value, adj.P.Val: normalized p-value, and B: log Odds ratio. (PDF) [file pone.0052718.s005.pdf]

Table S4

down-regulated

GeneName	Description	logFC	AveExpr	t	P.Value	adj.P.Val	B
BCL2	Homo sapiens B-cell CLL/lymphoma 2 (BCL2), nuclear gene encoding mitochondrial protein, transcript variant alpha, mRNA [NM_000633]	-0,90344777	8,53039694	-3,47576717	0,00843817	0,05308792	-2,62544526
		-0,92655385	8,672236	-3,42533016	0,00908645	0,05522161	-2,70080958
		-0,92938473	8,75080072	-3,42059262	0,00915	0,05543568	-2,70790236
		-0,94326091	8,83172924	-3,54377158	0,00764074	0,05035931	-2,52427232
		-0,94502781	8,80800164	-3,55487682	0,00751832	0,04992194	-2,50780037
		-0,94985561	9,07413635	-3,43706237	0,00893108	0,05471184	-2,68325488
		-0,95042686	8,8999137	-3,46493503	0,00857314	0,0534589	-2,64160795
		-0,95356793	8,85331064	-3,42713897	0,00906231	0,05514936	-2,69810216
		-0,95822537	8,74513965	-3,5790245	0,00725925	0,04890381	-2,47203254
		-0,98300623	8,81461678	-3,44022721	0,00888965	0,05460801	-2,67852186
BIRC2	Homo sapiens baculoviral IAP repeat-containing 2 (BIRC2), mRNA [NM_001166]	-0,02957605	10,3794847	-0,37689705	0,71609997	0,82260998	-6,57428612
CDH18	Homo sapiens cadherin 18, type 2 (CDH18), mRNA [NM_004934]	-2,12314849	6,56519943	-3,58510842	0,00719549	0,04870021	-2,46303175
CDH19	Homo sapiens cadherin 19, type 2 (CDH19), mRNA [NM_021153]	-0,27046416	5,33720785	-1,45470257	0,1840241	0,34205323	-5,61551222
		-2,1464839	7,5091709	-3,32359904	0,01055997	0,05979331	-2,85361274
COCH	Homo sapiens coagulation factor C homolog, cochlin (Limulus polyphemus) (COCH), mRNA [NM_004086]	-4,07398058	11,7216186	-6,7118245	0,00015441	0,01103418	1,44543004
COCH	Homo sapiens coagulation factor C homolog, cochlin (Limulus polyphemus) (COCH), mRNA [NM_004086]	-4,5683394	11,303063	-5,95017337	0,00034875	0,01380795	0,62777457
FAM107A	Homo sapiens family with sequence similarity 107, member A (FAM107A), transcript variant 1, mRNA [NM_007177]	-0,76120136	6,45404292	-5,06869322	0,00098176	0,01922558	-0,42414801
		-1,57844616	9,7347994	-5,37529956	0,00067682	0,01688038	-0,04483397
		-1,69483244	9,42888563	-4,96580549	0,00111557	0,02018211	-0,55471075
FGFBP2	Homo sapiens fibroblast growth factor binding protein 2 (FGFBP2), mRNA [NM_031950]	-1,08048127	6,28107683	-3,40462826	0,00936763	0,0561588	-2,73182041
		-2,36661007	7,80059881	-4,10769357	0,00343877	0,03318956	-1,70776901
ID4	Homo sapiens inhibitor of DNA binding 4, dominant negative helix-loop-helix protein (ID4), mRNA [NM_001546]	-1,53152329	11,5609003	-6,32838755	0,0002307	0,01221771	1,04388452
PAX3	Homo sapiens paired box gene 3 (Waardenburg syndrome 1) (PAX3), transcript variant PAX3B, mRNA [NM_013942]	-0,21952212	5,66046168	-2,26226603	0,05369658	0,15457897	-4,47478251
		-0,32041205	5,53480269	-3,58712306	0,00717451	0,04863132	-2,46005218
		-2,04113543	7,11227155	-5,79562474	0,00041503	0,01427856	0,45179855
PRPH	Homo sapiens peripherin (PRPH), mRNA [NM_006262]	-1,19099548	7,6707356	-4,87643413	0,00124805	0,02119555	-0,66946521
SP5	Homo sapiens Sp5 transcription factor (SP5), mRNA [NM_001003845]	-1,98004418	7,64173115	-5,19678908	0,00083912	0,01820253	-0,26390367
		-2,48144498	7,74415062	-5,59898007	0,0005202	0,01532709	0,22277288

TFAP2B	Homo sapiens transcription factor AP-2 beta (activating enhancer binding protein 2 beta) (TFAP2B), mRNA [NM_003221]	-2,02858922	9,97566223	-3,6297085	0,0067459	0,04709474	-2,39718338
